# Arabinoxylan and Pectin Metabolism in Crohn’s Disease Microbiota: An *In Silico* Study

**DOI:** 10.3390/ijms23137093

**Published:** 2022-06-25

**Authors:** Carlos Sabater, Inés Calvete-Torre, Lorena Ruiz, Abelardo Margolles

**Affiliations:** 1 Department of Microbiology and Biochemistry of Dairy Products, Instituto de Productos Lácteos de Asturias-Consejo Superior de Investigaciones Científicas (IPLA-CSIC), Paseo Río Linares s/n, 33300 Villaviciosa, Asturias, Spain; ines.calvete@ipla.csic.es (I.C.-T.); lorena.ruiz@ipla.csic.es (L.R.); amargolles@ipla.csic.es (A.M.); 2 Functionality and Ecology of Beneficial Microbes (MicroHealth) Group, Instituto de Investigación Sanitaria del Principado de Asturias (ISPA), 33011 Oviedo, Asturias, Spain

**Keywords:** arabinoxylan, pectin, metagenome-assembled genomes, carbohydrate metabolism, Crohn’s disease, cross-feeding

## Abstract

Inflammatory bowel disease is a chronic disorder including ulcerative colitis and Crohn’s disease (CD). Gut dysbiosis is often associated with CD, and metagenomics allows a better understanding of the microbial communities involved. The objective of this study was to reconstruct *in silico* carbohydrate metabolic capabilities from metagenome-assembled genomes (MAGs) obtained from healthy and CD individuals. This computational method was developed as a mean to aid rationally designed prebiotic interventions to rebalance CD dysbiosis, with a focus on metabolism of emergent prebiotics derived from arabinoxylan and pectin. Up to 1196 and 1577 MAGs were recovered from CD and healthy people, respectively. MAGs of *Akkermansia muciniphila*, *Barnesiella viscericola* DSM 18177 and *Paraprevotella xylaniphila* YIT 11841 showed a wide range of unique and specific enzymes acting on arabinoxylan and pectin. These glycosidases were also found in MAGs recovered from CD patients. Interestingly, these arabinoxylan and pectin degraders are predicted to exhibit metabolic interactions with other gut microbes reduced in CD. Thus, administration of arabinoxylan and pectin may ameliorate dysbiosis in CD by promoting species with key metabolic functions, capable of cross-feeding other beneficial species. These computational methods may be of special interest for the rational design of prebiotic ingredients targeting at CD.

## 1. Introduction

Inflammatory bowel disease (IBD) is a chronic and relapsing inflammatory disorder of the intestine with a multifactorial etiology. IBD mainly includes ulcerative colitis and Crohn’s disease (CD) and its prevalence has been increasing worldwide [1,2]. It has been estimated that more than 1.5 million people in the United States and 2 million people in Europe suffer from IBD [3]. The progression of IBD involves complex interactions between the immune system, human microbiota and the environment [2,4]. In this regard, it is believed that dysbiosis of the human gut microbiota exacerbates IBD symptoms [4]. In this sense, metagenomic sequencing has been used to decipher alterations of the taxonomic composition and metabolic profiles of IBD microbiota as a mean to identify biomarkers of disease and targets for intervention [1]. However, metagenomics also allows recovering complete metagenome-assembled genomes (MAGs) from complex microbial communities and large datasets, which can provide valuable information on the metabolic potential of specific members of the community [5,6,7]. In addition, bioinformatics methods to analyse MAGs sequences in order to explore synergistic interactions between gut microbes have been developed [8]. In this regard, potential cross-feeding mechanisms between these gut bacteria can be elucidated *in silico*. Cross-feeding reduces competition between gut microbes and enhances their growth. In this sense, essential symbionts and alternative symbionts involved in cross-feeding interactions can be determined. Essential symbionts comprise key microorganisms that occur in every minimal community of host MAGs needed to satisfy one specific metabolic function through metabolic cooperation of these bacteria. These functions involve the metabolisation of different compounds present in colon lumen. In contrast, alternative symbionts occur only in some of these minimal communities of cross-feeders. Therefore, any of the alternative symbionts can complete the missing metabolic functions of the minimal microbial community. These biologically relevant data cannot be obtained following assembly-free methods. However, the majority of studies have focused on the recovery of MAGs from healthy microbiota samples, and few have explored their potential to aid and reconstruct metabolic capacities in both health and dysbiosis states, with the final aim to assist the rational design of prebiotic interventions targeting gut microbiota modulation in specific population groups.

Genome annotation of MAGs can be used to study specific enzymatic activities such as those involved in carbohydrate metabolism [9]. Furthermore, MAGs could be used to build advanced genome-scale metabolic networks. These metabolic models reduce the complexity of large-scale microbiota into minimal communities with equivalent metabolic properties [8,10]. For example, genome-scale metabolic modelling revealed differential patterns in the bile acid metabolism of microbiomes of pediatric IBD patients [11]. To our knowledge, no previous attempts to elucidate symbiotic relationships in IBD microbiota and their influence on other metabolic activities such as its polysaccharide fermentation capability have been reported. In this regard, it has been described that the interaction of diet and the gut microbiota is perturbed in patients with IBD [12], while the intake of low fiber diets results in gut dysbiosis and promotes inflammation of the gut [12,13]. In contrast, pro- and prebiotic administration enhances the effectivity of IBD treatment [2]. Specifically, probiotic microorganisms belonging to *Bifidobacterium* and *Lactobacillus* genera may be beneficial for IBD remission. It should be considered that vegetable poly- and oligosaccharides comprise different families such as arabinoxylan and pectin-derived compounds that have been proposed as emerging prebiotics with enhanced bioactivity [14]. These prebiotic mixtures could be tailored to target specific diseases such as IBD [15,16]. In addition, previous studies suggest the beneficial effects of pectin, arabinoxylan and oligosaccharides derived from these substrates to ameliorate IBD symptoms in clinical research [17,18,19].

Arabinoxylan consist of a linear backbone of 1500 to 15,000 β(1-4) D-xylopyranoside units, which can occur substituted with α-L-arabinofuranoside residues positioned on C-(*O*)-2 or C-(*O*)-3 [14,20]. In addition, xylose monomers may be substituted with glucuronic acid and its 4-O-methyl derivative. On the other hand, pectin is mainly composed of linear chains of α-1,4-D-galacturonic acid (GalA) called homogalacturonan. Moreover, ramified domains of pectin comprise alternate sequences of GalA and α-(1, 2) linked α-L-rhamnosyl residues, which may be substituted at *O*-4 with linear or branched oligosaccharides [14,21]. Therefore, microbial enzymes acting on these polysaccharide structures involved the following activities: arabinofuranosidases, xylosidases, xylanases, xylan glucuronidases and oxidases, acetyl xylan esterases and pectin esterases, polygalacturonases, rhamnosidases, and polygalacturonate and rhamnogalacturonan lyases. As an approach to aid design prebiotics based on arabinoxylan or pectin, targeted to ameliorate IBD gut dysbiosis, microbial enzymes involving these domains should be investigated.

Therefore, the aim of this study was to employ several computational methods to reconstruct *in silico* carbohydrate metabolic capabilities from MAGs recovered from the microbiota of both healthy and CD individuals. Glycosidase profiles and potential metabolic activity of gut microbes from healthy and CD microbiota have been compared. This bioinformatics approach was developed as a mean to aid rationally designed prebiotic interventions to rebalance the CD dysbiosis, with a focus on the metabolism of emergent prebiotics derived from arabinoxylan and pectin.

## 2. Results and Discussion

### 2.1. MAGs Recovery

**Figure 1:**
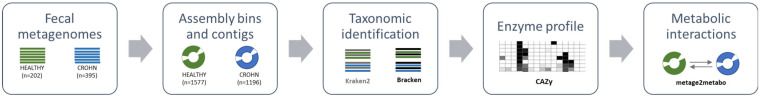
Computational workflow used in this work. Metagenome-assembled genomes (MAGs) were recovered from the microbiota of patients with Crohn’s disease and healthy individuals. Taxonomic identification of MAGs was performed using Kraken2 and Bracken software. MAGs sequences were mapped against the Carbohydrate-Active enZYmes Database (CAZy) to annotate glycosidase domains. Finally, metabolic interactions between MAGs were elucidated using metage2metabo software (v. 1.5.0) developed by Belcour et al. [8].

Metabolic capabilities of gut microbes present in the microbiota of patients with CD and healthy individuals have been studied. Specifically, glycolytic activities of gut microbes and their synergistic interactions in the presence of pectin and arabinoxylan have been investigated *in silico*. Figure 1 illustrates the computational workflow used in this work. A total 1196 and 1577 MAGs were first recovered from CD and healthy fecal samples, respectively (Table 1 and Table 2, Supplementary material Tables S1 and S2). As it can be seen, some of these MAGs could be identified at strain or species level while other sequences could be correctly identified only at genus or family level. Interestingly, several microbial clades (total number of different taxa *n* = 41) were also found in the microbiota of both patients with CD and healthy individuals (Table 1 and Table 2). In this regard, these common taxa include health-promoting genera such as *Faecalibacterium, Akkermansia, Blautia* and *Paraprevotella* [14,22].

MAGs from a few clades were exclusively recovered from one of the studied groups. MAGs from the clades *Parabacteroides distasonis*, *Roseburia intestinalis* L1-82, *Veillonella*, *Clostridium bolteae* and *Muribaculum* sp. TLL-A4 and other gut microbes were exclusively retrieved from IBD metagenomes (total number of different taxa *n* = 25, Supplementary material Table S1), whereas MAGs from unidentified species of *Parabacteroides*, *Clostridium*, *Roseburia*, *Muribaculum*, as well as *B. adolescentis* ATCC 15703 and other species were only retrieved from healthy metagenomes (total number of different taxa *n* = 42, Supplementary material Table S2).

As explained, several bacterial genera were found in both IBD and healthy metagenomes. These MAGs belong to common host species from the human colon. It has been reported that Lachnospiraceae were not significantly increased in patients with IBD [23]. In the present study, a similar number of MAGs of unidentified members of Lachnospiraceae and other genera belonging to this family such as *Blautia* were recovered from IBD patients and healthy participants. This microbial family belong to the healthy core gut microbiota and encompass the main producers of short-chain fatty acids (SCFAs). In addition, IBD typically involves a reduction in microbial diversity and the lower abundance of Firmicutes [1]. A higher number of MAGs of Firmicutes such as *Ruminococcus*, were recovered from healthy participants than from IBD patients (Table 1 and Table 2). On the other hand, MAGs of gut microbes affected by CD dysbiosis, according to the literature, were obtained from both types of samples (healthy participants and CD patients) (Table 1 and Table 2). In this regard, it has been described that IBD results in a decrease of bacteria with anti-inflammatory capacities such as *F. prausnitzii* when compared to healthy participants [1]. This species belonging to *Clostridium* cluster IV induces the proliferation of regulatory T cells associated with IBD. *F. prausnitzii* also exerts an anti-inflammatory effect by producing butyrate and microbial anti-inflammatory molecules [15,24]. A decrease in the concentration of SCFAs produced by *F. prausnitzii* and *Clostridium* clusters IV, XIVa and XVIII has been found in IBD patients [25]. Similarly, several species belonging to *Blautia*, *Clostridium*, *Roseburia* and *Ruminococcus* are decreased in patients with CD [1], including children, adolescents and adults [1,23]. On the contrary, increases in Proteobacteria, mainly *E. coli*, and Bacteroidetes have been reported in patients with CD when compared to healthy subjects [1,26,27].

Some microbial clades identified in IBD and healthy microbiota such as *A. muciniphila* and *F. prausnitzii* comprise specialist primary degrading gut anaerobes involved in the breakdown of dietary fiber [28], leading to SCFAs production and health benefits [15]. Therefore, it is of great interest to study the carbohydrate metabolism of these microorganisms in order to design IBD targeting prebiotics.

### 2.2. Study of MAGs Glycosidases Acting on Arabinoxylan and Pectin

**Figure 2:**
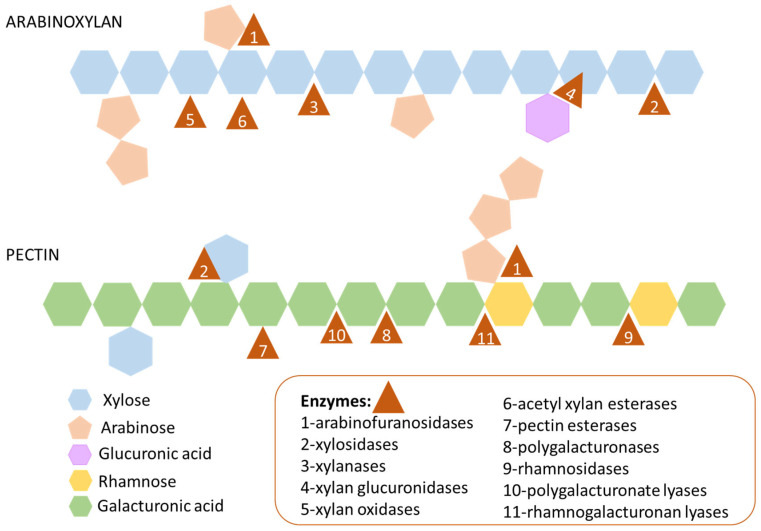
Schematic representation of enzyme domains acting on arabinoxylan and pectin that were annotated in metagenome-assembled genomes (MAGs) recovered from the microbiota of patients with Crohn’s disease and healthy individuals.

**Figure 3:**
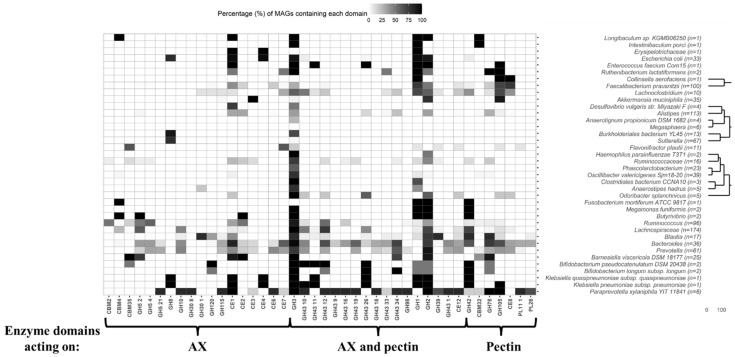
Heatmap showing the presence of different glycosidases (indicated as black cells) in metagenome-assembled genomes (MAGs) recovered from the microbiota of patients with Crohn’s disease. These MAGs were assigned to taxonomic clades that were also found in the microbiota of healthy individuals (see Table 1). The percentage (%) of MAGs containing each functional domain is shown. Specifically, glycosidases capable of degrading arabinoxylan (AX) and pectin are illustrated. Glycosidase functional domains showing coverage values higher than 0.95 were annotated. Codes corresponding to the Carbohydrate-Active enZYmes Database (CAZy) family of each enzyme have been assigned.

**Figure 4:**
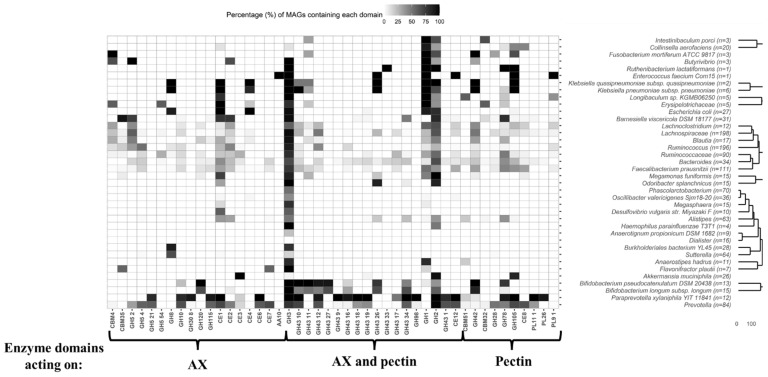
Heatmap showing the presence of different glycosidases (indicated as black cells) in metagenome-assembled genomes (MAGs) recovered from the microbiota of healthy individuals. These MAGs were assigned to taxonomic clades that were also found in the microbiota of patients with Crohn’s disease (see Table 2). The percentage (%) of MAGs containing each functional domain is shown. Specifically, glycosidases capable of degrading arabinoxylan (AX) and pectin are illustrated. Glycosidase functional domains showing coverage values higher than 0.95 were annotated. Codes corresponding to the Carbohydrate-Active enZYmes Database (CAZy) family of each enzyme have been assigned.

In order to deepen the carbohydrate metabolism potential of MAGs from gut microbes retrieved from CD and healthy individual’s metagenomes, glycosidase analysis was performed. Specifically, characteristic profiles of glycosidases acting on arabinoxylan and pectin chains were investigated (Figure 2, Figure 3 and Figure 4, Supplementary material Tables S3–S6, Supplementary material Figures S1 and S2). A schematic representation of enzyme domains acting on arabinoxylan and pectin is provided in Figure 2. As an approach to aid design prebiotics based on arabinoxylan or pectin, targeted to ameliorate IBD gut dysbiosis, first the enzyme machinery of the MAGs recovered from both IBD and healthy gut metagenomes to hydrolyse these carbohydrates was investigated.

*Arabinoxylan degrading enzymes.* Arabinoxylan degrading enzymes were annotated in several MAGs of a wide range of genera present in both IBD and healthy metagenomes including *Anaerostipes*, *Bacteroides*, *Bifidobacterium*, *Blautia*, *Butyrivibrio,* *Faecalibacterium,* unidentified members of Lachnospiraceae, *Prevotella*, *Paraprevotella* and *Ruminococcus* (Figure 3 and Figure 4, Supplementary material Tables S3 and S4). The potential of several of these genera to metabolise emerging prebiotics was reported in a previous study [14]. With regard to CD samples, xylanase domains were identified in all MAGs of *Butyrivibrio* and *B. viscericola* DSM 18177, as well as in most MAGs of other gut microbes such as *P. xylaniphila* YIT 11841 (Figure 3, Supplementary material Table S3). Similar results were obtained for healthy microbiota samples (Figure 4, Supplementary material Table S4). Xylosidase domains were annotated in all MAGs of *B. pseudocatenulatum* DSM 20438 recovered from healthy microbiota samples, while xylan glucuronidases were identified in the majority of MAGs of *P. xylaniphila* YIT 11841 recovered from IBD and healthy metagenomes (Figure 3 and Figure 4, Supplementary material Tables S3 and S4). Other functional domains of interest involve acetyl xylan esterases identified in most MAGs of *B. viscericola* DSM 18177 and *P. xylaniphila* YIT 11841 recovered from IBD metagenomes (Figure 3, Supplementary material Table S3). These domains were also annotated in several MAGs recovered from healthy metagenomes (Figure 4, Supplementary material Table S4). In contrast, xylan oxidase domains were only found in MAGs of *E. faecium* Com15 recovered from healthy metagenomes (Figure 4, Supplementary material Table S4).

*Arabinoxylan and pectin degrading enzymes.* Enzyme domains involving both xylanases and arabinofuranosidases were present in the majority of MAGs of several clades including *Bacteroides*, Lachnospiraceae, *Ruminococcus, B. viscericola* DSM 18177, *O. splanchnicus* and *P. xylaniphila* YIT 11841 recovered from IBD and healthy metagenomes (Figure 3 and Figure 4, Supplementary material Tables S3 and S4). It has been reported that arabinose residues are metabolised by a limited amount of gut microbes containing arabinofuranoside activities. In addition, few microbial species show full xylanolytic capacity so arabinoxylan are first hydrolysed by first degraders such as specific *Bacteroides* species. Hydrolysis products are then utilised by other bacteria that cannot grow on xylan, leading to cross-feeding interactions [14,29]. Unspecific domains comprising arabinofuranosidases, β-galactosidases and β-xylosidases were identified in most MAGs of *B. viscericola* DSM 18177 recovered from patients with CD (Figure 3, Supplementary material Table S3). Other domains involving multiple activities of interest such as acetyl xylan esterase and pectin acetylesterase were mainly found in MAGs of *P. xylaniphila* YIT 11841 recovered from IBD and healthy metagenomes (Figure 3 and Figure 4, Supplementary material Tables S3 and S4).

*Pectin degrading enzymes.* With regard to functional profiles of glycosidases acting on pectin, rhamnosidases were annotated in the majority of MAGs of *A. muciniphila*, *B. viscericola* DSM 18177 and *P. xylaniphila* YIT 11841 from IBD and healthy participants (Figure 3 and Figure 4, Supplementary material Tables S3 and S4). In addition, rhamnogalacturonan lyases were found in most MAGs of *P. xylaniphila* YIT 11841 recovered from IBD and healthy metagenomes (Figure 3 and Figure 4, Supplementary material Tables S3 and S4).

Some glycosidases were identified only in MAGs recovered from metagenomes from patients with CD. It should be noted that these glycosidases were annotated in a limited number of MAGs belonging to the same clade, highlighting the role of metabolic variability between strains (Supplementary material Figure S1 and Table S5). On the other hand, several functional domains were found only in MAGs recovered from healthy individuals comprising starch-binding enzymes and cellulases (Supplementary material Figure S2 and Table S6). These activities were similar to those only identified in IBD metagenomes (Supplementary material Figure S1 and Table S5).

A wide number of characteristic glycosidase domains acting on arabinoxylan and pectin were annotated in MAGs of *A. muciniphila*, *B. viscericola* DSM 18177 and *P. xylaniphila* YIT 11841 recovered from metagenomic samples of patients with CD and healthy individuals. These unique enzymatic activities involved xylan glucuronidases, acetyl xylan esterases, pectin acetylesterases, rhamnosidases and rhamnogalacturonan lyases that were not found in MAGs of other gut microbes. These results suggest that metabolic activities of these species might not be greatly affected by gut dysbiosis in CD. Therefore, the rational design of prebiotics derived from arabinoxylan and pectin could be of special interest to ameliorate CD symptoms through its metabolism by these core species. Previous studies suggest that administration of arabinoxylan oligosaccharides and high methoxylated pectin increased the abundance of *A. muciniphila* [30]. Similarly, prebiotic administration of a highly branched rhamnogalacturonan type I-enriched pectin increased the abundance of *Barnesiella* and other beneficial genera such as *Butyrivibrio*, *Roseburia*, *Flavonifractor*, *Acetivibrio* and *Clostridium* cluster IV [31]. Moreover, the ability of *Paraprevotella* genus to metabolise xylan and pectin leading to SCFAs production has been described [22].

### 2.3. Cross-Feeding between MAGs in the Presence of Arabinoxylan and Pectin

**Figure 5:**
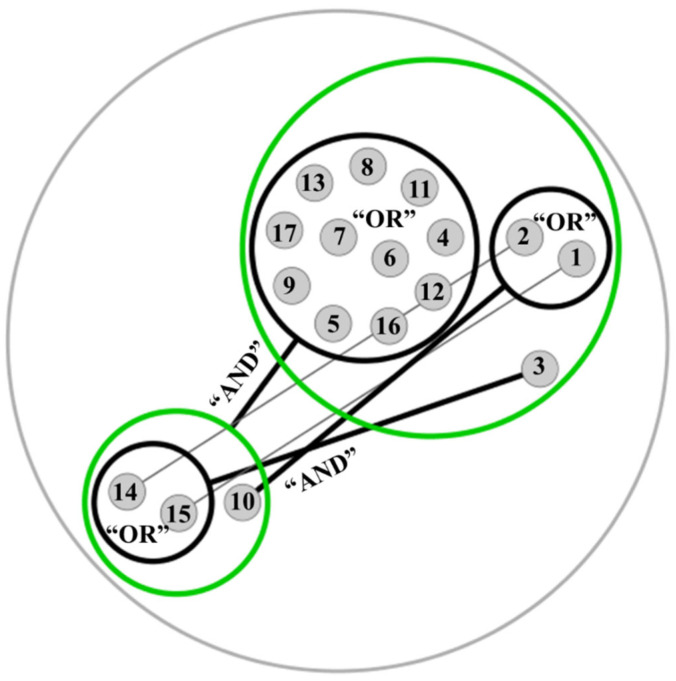
Metabolic network illustrating potential cross-feeding mechanisms between metagenome-assembled genomes (MAGs) recovered from the microbiota of healthy individuals in the presence of pectin. These MAGs involve beneficial arabinoxylan and pectin degraders (*Akkermansia muciniphila*, *Barnesiella viscericola* DSM 18177, *Paraprevotella xylaniphila* YIT 11841) that were also found in the microbiota of patients with Crohn’s disease (see Figure 1). In addition, MAGs of other microorganisms that were not identified in the microbiota of patients with Crohn’s disease and might be greatly affected by gut dysbiosis were also chosen. Network nodes (i.e., circles containing different microbial communities showing equivalent metabolic functions) are connected by black lines indicating synergistic relationships between communities and complementary metabolic functions. Metabolic functions of MAGs from different nodes are needed to achieve the maximum number of end products from pectin as well as other colonic metabolites (this mutualistic relationship is indicated by the conjunction “AND”). MAGs inside the same node play the same role and could be replaced by other members from the same community (this similar role is indicated by the conjunction “OR”). (**1**): *A. muciniphila*, (**2**): *B. viscericola* DSM 18177, (**3**): *P. xylaniphila* YIT 11841, (**4**): unidentified *Parabacteroides* species, (**5**): unidentified *Roseburia* species, (**6**): *Bifidobacterium adolescentis* ATCC 15703, (**7**): *Acidaminococcus fermentans* DSM 20731,(**8**): *Brachyspira murdochii* DSM 12563, (**9**): *Desulfovibrio piger*, (**10**): *Faecalitalea cylindroides* T2-87, (**11**): *Lactobacillus ruminis* ATCC 27782, (**12**): *Methanobrevibacter smithii* ATCC 35061, (**13**): *Streptococcus thermophilus*, (**14**): *Veillonella atypica*, (**15**): *Clostridioides difficile*, (**16**): *Lactobacillus mucosae* LM1, (**17**): *Veillonella parvula* HSIVP1.

To gain a better understanding on the ecological impact of MAGs showing glycosidase domains acting on arabinoxylan and pectin to sustain gut microbiota ecosystem networks, potential cross-feeding mechanisms between these bacteria were elucidated *in silico* according to Belcour et al. [8]. The number of genes associated to different microbial metabolic activities annotated in MAGs ranged from 361 to 2074 on IBD samples, and from 315 to 2080 on samples from healthy individuals. This parameter was calculated according to Belcour et al. [8]. To illustrate potential cross-feeding mechanisms between different taxa of interest, arabinoxylan and pectin degraders (*A. muciniphila*, *B. viscericola* DSM 18177 and *P. xylaniphila* YIT 11841) found in the microbiota of both healthy participants and patients with CD, were selected. These species and strains showed a wide range of characteristic enzyme activities acting on arabinoxylan and pectin (i.e., xylan glucuronidases and xylan esterases, pectin acetylesterases, rhamnosidases and rhamnogalacturonan lyases) that were not found in other gut microbes (see Results and Discussion Section 2.2.). These three clades have been associated to several health benefits including SCFAs production [22,30,31]. On the other hand, MAGs of other microorganisms that were identified only in the microbiota of healthy individuals (i.e. these MAGs were not recovered from the microbiota of patients with CD, Supplementary material Table S2) were included in the metabolic interaction study (Figure 5). These clades might be greatly affected by gut dysbiosis. To compute the metabolic network, one MAG per clade showing the highest number of genes associated to metabolite production was chosen.

Several cross-feeding mechanisms were observed among pectin and arabinoxylan degraders (*A. muciniphila*, *B. viscericola* DSM 18177 and *P. xylaniphila* YIT 11841) and those clades affected by gut dysbiosis (i.e., those that could only be recovered from healthy individuals) (Figure 5). Similar metabolic interactions were determined in the presence of pectin and arabinoxylan (Figure 5, Supplementary material Figure S3). *A. muciniphila* and *B. viscericola* DSM 18177 showed equivalent metabolic functions and potential synergistic relationships with *F. cylindroides* T2-87. On the other hand, cross-feeding mechanisms between *P. xylaniphila* YIT 11841 and *C. difficile* or *V. atypica* were elucidated. Interestingly, *F. cylindroides* T2-87, *C. difficile* and *V. atypica* showed potential cross-feeding mechanisms with the rest of gut microbes affected by gut dysbiosis (Figure 5).

These results suggest that *A. muciniphila*, *B. viscericola* DSM 18177 and *P. xylaniphila* YIT 11841 may establish metabolic interactions with gut microbes reduced in CD. This fact could be attributed to key functions associated to arabinoxylan and pectin metabolism due to their characteristic enzyme machinery (Figure 3 and Figure 4). Therefore, administration of prebiotic mixtures derived from arabinoxylan and pectin [14], as well as symbiotic combinations of these prebiotics and cross-feeder bacteria reduced in CD may result in the amelioration of gut dysbiosis.

It should be taken into account that one of the limitations of prebiotic supplementation in CD patients is the potential increment in gas production associated with excessive fermentation. In this sense, ingestion of traditional prebiotics such as FOS may be associated with gastrointestinal symptoms including abdominal bloating and flatulence. Clinical trials demonstrated that high-dose inulin-type fructans prebiotics may actually worsen CD symptoms [32,33]. However, doses of 15 g/day of fructo-oligosaccharides (FOS) were well tolerated in patients with CD and led to a significant reduction in the disease activity [32]. Taking into account these limitations, computational models described in the present work allow predicting the number of microbial taxa involved in gas formation, although gas concentrations cannot be predicted by this pipeline. In this regard, no major differences were observed in the number of taxa involved in hydrogen production between microbial cross-feeding simulations in the presence of pectin, arabinoxylan and without prebiotics. It was found that up to 337 MAGs recovered from CD patients corresponding to 51 different taxa might contribute to hydrogen production. It should be considered that the amount of gas produced cannot be calculated by the models. Therefore, the suitability of pectin and arabinoxylan-derived compounds as prebiotics targeting at CD should be validated in future clinical studies.

## 3. Materials and Methods

### 3.1. Metagenome Selection

To investigate arabinoxylan and pectin metabolism by gut microbiota in patients with CD and healthy individuals, curated human metagenomes were retrieved using the standardised database HumanMetagenomeDB [34]. Unassembled reads produced by an Illumina platform showing a minimum sequencing depth of 10 million reads were selected. As a result, CD fecal metagenomes (*n* = 395) from three sequencing experiments [35,36,37] were retrieved: BioProject identity (ID) 46321 (sequence library IDs SRR495449-SRR497947), BioProject ID 321058 (sequence library IDs SRR3582131-SRR3582183) and BioProject ID 398089 (sequence library IDs SRR5935744-SRR5962905). These samples corresponded to fecal metagenomes from male and female participants from the United States of America belonging to the following age groups: children, teenagers, adults and elders. On the other hand, healthy metagenomes (*n* = 202) used as controls were retrieved from nine different studies and reference datasets [38,39,40,41,42,43,44,45,46]: BioProject ID 266076 (sequence library IDs ERR478964-ERR479598), BioProject ID 305507 (sequence library IDs SRR2992882-SRR2992961), BioProject ID 356544 (sequence library IDs SRR5088933-SRR5088943), BioProject ID 382085 (sequence library IDs ERR1912957-ERR1913126), BioProject ID 356102 (sequence library IDs SRR5091454-SRR5091619), BioProject ID 388263 (sequence library IDs SRR5813226-SRR5813545), BioProject ID 397664 (sequence library IDs SRR5925338-SRR5925343), BioProject ID 492716 (sequence library IDs SRR8113241-SRR8113264) and BioProject ID 520750 (sequence library IDs DRR127488-DRR162776). These samples corresponded to fecal metagenomes from male and female participants from several countries (Australia, Canada, China, France, Germany, United States of America and Japan) belonging to the adult and elder age groups.

### 3.2. Metagenome Assembly

MAGs were recovered according to the method described by Sabater et al. [9], using validated workflows for metagenome assembly and taxonomic classification [5]. These computational pipelines were used to process metagenomes from patients with CD and healthy individuals to further compare their metabolic activities (Figure 1).

Contaminant reads and low-quality sequences were separated *in silico* from microbial reads using Kneaddata (v0.7.4) and Trimmomatic (v0.39) software. For this purpose, minimum length of output reads was computed as 50 percent of the length of the input reads considering a sliding window of 4:20. Bowtie2 (v.2.4.2) was used to map metagenomic reads [47], against the reference databases Homo sapiens hg37 and human contamination Bowtie2 (v.2.3.5.1) in order to remove host contamination. Metagenome sequences were assembled using MEGAHIT v.1.2.9 software with default settings [48]. Maximum k-mer size was set at 127 in order to generate the following series of k-mers with lengths shorter than entire reads (k-21, k-31, k-41, k-51, k-61, k-71, k-81, k-91, k-101, k-111, k-121, k-127). Metagenome reads were mapped against the assembly using Bowtie2 [47]. Output bam files generated were sorted and indexed. Metagenome binning of contigs larger than 1.5 kilobases was performed using MetaBAT2 v.2.2.15 software with default settings [49]. Once MAGs were obtained, CheckM v.1.1.3 lineage-specific workflow was run to assess their completeness and contamination [50]. Quality standards of MAGs were established according to Asnicar et al. [5]. MAGs showing completeness lower than 50% and contamination higher than 5% were discarded. Taxonomic classification of pruned MAGs at family, species genus and strain level, was performed following Kraken2 standard workflow [51]. Then, these taxonomic assignments were curated using Bracken software (Bayesian Reestimation of Abundance with Kraken) [52]. This software uses the taxonomic assignments made by Kraken2, along with information about the genomes themselves to accurately classify sequences even when a sample contains multiple near-identical species.

### 3.3. In Silico Study of Arabinoxylan and Pectin Metabolism

Metabolic capacity of MAGs obtained from patients with CD and healthy individuals to degrade arabinoxylan and pectin was assessed through comparative genomics. First, glycosidase functional domains of MAGs were annotated following “run_dbcan” pipeline developed by Zhang et al. [53]. To this aim, MAGs sequences were mapped against the Carbohydrate-Active enZYmes Database (CAZy, http://www.cazy.org/last accessed on 23 February 2022). To identify functional domains of interest (i.e., glycosidase domains acting on arabinoxylan and pectin chains), HMMER software for biosequence analysis [54], and Pfam database [55], were used. Coverage values higher than 0.95 were considered to provide high-quality annotated glycosidase sequences. Metabolic capabilities of MAGs were compared through hierarchical clustering calculated by the complete linkage method. In the complete linkage method, all pairwise dissimilarities between the elements in each cluster (i.e., glycosidase domains of MAGs acting on arabinoxylan and pectin) are calculated using the basic function “hclust” from R (v.3.6.2). Then, the largest dissimilarity value is chosen as the distance between clusters to yield more compact clusters.

Once complete glycosidase profiles of MAGs were elucidated, potential symbiotic relationships between MAGs from patients with CD and healthy individuals were studied *in silico*. For this purpose, MAGs were annotated using Prokka pipeline [56]. Then, standard Genbank files (.gbk) files containing sequences and annotations obtained by Prokka were selected to elucidate potential cross-feeding interaction mechanisms using metage2metabo software [8]. A set of nutrients (seeds) that may be present in human gut according to Belcour et al. [8], was provided to the algorithm in order to calculate potential metabolic networks in given nutritional conditions. Different simulations were performed in the presence of arabinoxylan and pectin.

## 4. Conclusions

A total of 1196 and 1577 MAGs have been recovered from CD and healthy fecal samples, respectively. Glycosidase profiles of MAGs have been annotated and functional domains acting on pectin and arabinoxylan have been identified. A broad range of enzymes acting on arabinoxylan and pectin were annotated in MAGs of *A. muciniphila*, *B. viscericola* DSM 18177 and *P. xylaniphila* YIT 11841 recovered from metagenome sequencing data of patients with CD and healthy individuals. In addition, potential cross-feeding mechanisms between these taxa and other gut microbes not found in CD in the presence of arabinoxylan and pectin have been elucidated. As a result, we propose that arabinoxylan and pectin administration may ameliorate gut dysbiosis in CD by promoting taxa through cross-feeding network interactions with key commensal species. Computational simulations herein presented provide a first insight into the prediction of complex microbial interactions within the gut ecosystem and how these can guide the rational design of prebiotic/synbiotic preparations targeting CD dysbiosis.

## Figures and Tables

**Table 1:** Number of metagenome-assembled genomes (MAGs, *n* = 998) recovered from the microbiota of patients with Crohn’s disease and assigned to taxonomic clades that were also found in the microbiota of healthy individuals. MAGs were identified at family, genus, species or strain level. It should be noted that these common clades (total number of different taxa *n* = 41) were identical to those shown in Table 2.

MAGs Found in the Microbiota of Patients with Crohn’s Disease That were also Found in the Microbiota of Healthy Individuals	
**Taxa**	***n***
Lachnospiraceae	174
*Alistipes*	113
*Faecalibacterium prausnitzii*	100
*Ruminococcus*	96
*Sutterella*	67
*Prevotella*	61
*[Eubacterium] eligens* ATCC 27750	41
*Oscillibacter valericigenes* Sjm18–20	39
*Bacteroides*	36
*Akkermansia muciniphila*	35
*Escherichia coli*	33
*Barnesiella viscericola* DSM 18177	25
*Phascolarctobacterium*	23
*Acidaminococcus intestini* RyC-MR95	22
*Blautia*	17
Ruminococcaceae	16
Burkholderiales bacterium YL45	13
*Dialister*	13
*Flavonifractor plautii*	11
*Lachnoclostridium*	10
*Megasphaera*	6
*Paraprevotella xylaniphila* YIT 11841	6
*Anaerostipes hadrus*	5
*Odoribacter splanchnicus*	5
*Anaerotignum propionicum* DSM 1682	4
*Desulfovibrio vulgaris* str. Miyazaki F	4
Clostridiales bacterium CCNA10	3
*Bifidobacterium longum* subsp. *longum*	2
*Bifidobacterium pseudocatenulatum* DSM 20438 = JCM 1200 = LMG 10505	2
*Butyrivibrio*	2
*Haemophilus parainfluenzae* T3T1	2
*Megamonas funiformis*	2
*Ruthenibacterium lactatiformans*	2
*Collinsella aerofaciens*	1
*Enterococcus faecium* Com15	1
Erysipelotrichaceae	1
*Fusobacterium mortiferum* ATCC 9817	1
*Intestinibaculum porci*	1
*Klebsiella pneumoniae* subsp. *pneumoniae*	1
*Klebsiella quasipneumoniae* subsp. *quasipneumoniae*	1
*Longibaculum* sp. KGMB06250	1
**Total**	**998**

**Table 2:** Number of metagenome-assembled genomes (MAGs, *n* = 1361) recovered from the microbiota of healthy individuals and assigned to taxonomic clades that were also found in the microbiota of patients with Crohn’s disease. MAGs were identified at family, genus, species or strain level. It should be noted that these common clades (total number of different taxa *n* = 41) were identical to those shown in Table 1.

MAGs Found in the Microbiota of Healthy Individuals That Were also Found in the Microbiota of Patients with Crohn’s Disease	
**Taxa**	***n***
Lachnospiraceae	198
*Ruminococcus*	196
*Faecalibacterium prausnitzii*	111
Ruminococcaceae	90
*Prevotella*	84
*[Eubacterium] eligens* ATCC 27750	79
*Phascolarctobacterium*	70
*Sutterella*	64
*Alistipes*	63
*Oscillibacter valericigenes* Sjm18–20	36
*Bacteroides*	34
*Barnesiella viscericola* DSM 18177	31
Burkholderiales bacterium YL45	28
*Escherichia coli*	27
*Akkermansia muciniphila*	26
*Collinsella aerofaciens*	20
*Blautia*	17
*Dialister*	16
*Bifidobacterium longum* subsp. *longum*	15
*Megamonas funiformis*	15
*Megasphaera*	15
*Odoribacter splanchnicus*	15
*Bifidobacterium pseudocatenulatum* DSM 20438 = JCM 1200 = LMG 10505	13
*Lachnoclostridium*	12
*Paraprevotella xylaniphila* YIT 11841	12
*Anaerostipes hadrus*	11
*Desulfovibrio vulgaris* str. Miyazaki F	10
*Anaerotignum propionicum* DSM 1682	9
*Flavonifractor plautii*	7
*Klebsiella pneumoniae* subsp. *pneumoniae*	6
Erysipelotrichaceae	5
*Longibaculum* sp. KGMB06250	5
*Haemophilus parainfluenzae* T3T1	4
*Acidaminococcus intestini* RyC-MR95	3
*Butyrivibrio*	3
*Fusobacterium mortiferum* ATCC 9817	3
*Intestinibaculum porci*	3
*Klebsiella quasipneumoniae* subsp. *quasipneumoniae*	2
Clostridiales bacterium CCNA10	1
*Enterococcus faecium* Com15	1
*Ruthenibacterium lactatiformans*	1
**Total**	**1361**

## Supplementary Materials

The following supporting information can be downloaded at: https://www.mdpi.com/article/10.3390/ijms23137093/s1.
