# Supplementary material for: Arabinoxylan and Pectin Metabolism in Crohn’s Disease Microbiota: An In Silico Study
Source: Int J Mol Sci. 2022 Jun 25;23(13):7093. doi: 10.3390/ijms23137093 (PMC9266297; doi:10.3390/ijms23137093)
Supplement: Supplementary file 1 [file ijms-23-07093-s001.zip › ijms-1672093-supplementary.pdf]

International Journal of
Molecular Sciences

MDPI

Supplementary Materials

Arabinoxylan and Pectin Metabolism in Crohn's Disease Microbiota: An *In Silico* Study

Carlos Sabater [1,2,*], Inés Calvete-Torre [1,2], Lorena Ruiz [1,2] and Abelardo Margolles [1,2]

[1] Department of Microbiology and Biochemistry of Dairy Products, Instituto de Productos Lácteos de Asturias-Consejo Superior de Investigaciones Científicas (IPLA-CSIC), Paseo Río Linares s/n, 33300 Villaviciosa, Asturias, Spain; ines.calvete@ipla.csic.es (I.C.-T.); lorena.ruiz@ipla.csic.es (L.R.); amargolles@ipla.csic.es (A.M.)

[2] Functionality and Ecology of Beneficial Microbes (MicroHealth) Group, Instituto de Investigación Sanitaria del Principado de Asturias (ISPA), 33011 Oviedo, Asturias, Spain

* Correspondence: carlos.sabater@csic.es; Tel.: +34-985-892131

Table S1. Number of metagenome-assembled genomes (MAGs, n=198) recovered only from the mi-crobiota of patients with Crohn's disease. MAGs were identified at family, genus, species or strain level (total number of different taxa n=25).

**MAGs found only in the microbiota of patients with Crohn's disease**	
**Taxa**	**n**
Parabacteroides distasonis	62
Roseburia intestinalis L1-82	58
Veillonella	24
[Clostridium] bolteae	11
Muribaculum sp. TLL-A4	6
[Clostridium] saccharolyticum WM1	4
[Clostridium] sphenoides JCM 1415	4
Morganella morganii	4
Bacillaceae	3
Proteus mirabilis BB2000	3
Helicobacter canis	2
Klebsiella variicola	2
Proteus mirabilis HI4320	2
Roseburia hominis	2
[Clostridium] scindens ATCC 35704	1
Actinomyces sp. oral taxon 414	1
Bifidobacterium asteroides	1
Bifidobacterium dentium	1
Flintibacter sp. KGMB00164	1
Fusobacterium necrophorum subsp. *funduliforme*	1
Massilistercora timonensis	1
Muribaculum sp. H5	1
Olsenella sp. GAM18	1
Rhodobacteraceae	1
Streptococcus lutetiensis 033	1
**Total**	**198**

Table S2. Number of metagenome-assembled genomes (MAGs, n=216) recovered only from the mi-crobiota of healthy individuals. MAGs were identified at family, genus, species or strain level (total number of different taxa n=42).

**MAGs found only in the microbiota of healthy individuals**	
**Taxa**	**n**
Parabacteroides	54
Clostridium	34
Roseburia	20
Muribaculum	18
Bifidobacterium adolescentis ATCC 15703	14
Acidaminococcus fermentans DSM 20731	6
Brachyspira murdochii DSM 12563	5
Desulfovibrio piger	5
Faecalitalea cylindroides T2-87	5
Lactobacillus ruminis ATCC 27782	4
Methanobrevibacter smithii ATCC 35061	4
Streptococcus thermophilus	4
Veillonella atypica	4
Clostridioides difficile	3
Lactobacillus mucosae LM1	3
Veillonella parvula HSIVP1	3
Burkholderiaceae	2
Cutibacterium acnes	2
Lactobacillus fermentum	2
Turicibacter sp. H121	2
Absiella	1
Acidaminococcaceae	1
Adlercreutzia equolifaciens DSM 19450	1
Bifidobacterium animalis subsp. *animalis*	1
Bifidobacterium bifidum BGN4	1
Butyricimonas faecalis	1
Campylobacter concisus 13826	1
Clostridiales Family XIII. Incertae Sedis	1
Fusobacterium ulcerans ATCC 49185	1
Lactobacillus amylovorus DSM 20531	1
Lactobacillus salivarius str. Ren	1
Methanomassiliicoccaceae archaeon DOK	1
Muribaculaceae bacterium DSM 108610	1
Oscillibacter sp. PEA192	1
Oscillospiraceae	1
Paenibacillus	1
Rhodospirillaceae	1
Selenomonas sputigena ATCC 35185	1
Streptococcus salivarius JIM8777	1
Succinivibrio dextrinosolvens	1
Veillonella dispar	1
Victivallales bacterium CCUG 44730	1
**Total**	**216**

Table S3. Enzyme domains acting on arabinoxylan (AX) and pectin annotated in metagenome-assembled genomes (MAGs) recovered from the microbiota of patients with Crohn's disease. These MAGs were assigned to taxonomic clades that were also found in the microbiota of healthy individuals.

Domain	Acting on	Potential activities of interest
CBM2	AX	Xylanase
CBM4	AX	Xylanase
CBM35	AX	Xylanase
GH5 2	AX	Endo-β-1,4-xylanase
GH5 4	AX	Endo-β-1,4-xylanase
GH5 21	AX	Endo-β-1,4-xylanase
GH8	AX	Endo-β-1,4-xylanase
GH10	AX	Arabinoxylan-specific endo-β-1,4-xylanase
GH30 8	AX	Glucuronoarabinoxylan-specific endo-β-1,4-xylanase
GH30 1	AX	Endo-β-1,4-xylanase; β-xylosidase
GH120	AX	β-xylosidase
GH115	AX	Xylan α-1,2-glucuronidase
CE1	AX	Acetyl xylan esterase
CE2	AX	Acetyl xylan esterase
CE3	AX	Acetyl xylan esterase
CE4	AX	Acetyl xylan esterase
CE6	AX	Acetyl xylan esterase
CE7	AX	Acetyl xylan esterase
GH3	AX; pectin	Xylan 1,4-β-xylosidase; α-L-arabinofuranosidase
GH43 10	AX; pectin	Xylan 1,4-β-xylosidase; α-L-arabinofuranosidase
GH43 11	AX; pectin	Xylan 1,4-β-xylosidase; α-L-arabinofuranosidase
GH43 12	AX; pectin	Xylan 1,4-β-xylosidase; α-L-arabinofuranosidase
GH43 9	AX; pectin	α-L-arabinofuranosidase
GH43 16	AX; pectin	α-L-arabinofuranosidase
GH43 19	AX; pectin	α-L-arabinofuranosidase
GH43 26	AX; pectin	α-L-arabinofuranosidase
GH43 18	AX; pectin	α-L-arabinofuranosidase
GH43 33	AX; pectin	α-L-arabinofuranosidase
GH43 34	AX; pectin	β-D-galactofuranosidase; α-L-arabinofuranosidase
GH98	AX; pectin	Endo-β-1,4-galactosidase; endo-β-1,4-xylanase
GH1	AX; pectin	β-galactosidase; β-xylosidase
GH2	AX; pectin	β-galactosidase; α-L-arabinofuranosidase; β-xylosidase
GH39	AX; pectin	β-galactosidase; α-L-arabinofuranosidase; β-xylosidase
GH43 1	AX; pectin	β-galactosidase; α-L-arabinofuranosidase; β-xylosidase
CE12	AX; pectin	Acetyl xylan esterase; pectin acetylesterase

GH42	Pectin	β-galactosidase
CBM32	Pectin	Polygalacturonase
GH78	Pectin	α-L-rhamnosidase
GH105	Pectin	Unsaturated rhamnogalacturonyl hydrolase
CE8	Pectin	Pectin methylesterase
PL11 1	Pectin	Rhamnogalacturonan endo- / exolyase
PL26	Pectin	Rhamnogalacturonan exolyase

Table S4. Enzyme domains acting on arabinoxylan (AX) and pectin annotated in metagenome-assembled genomes (MAGs) recovered from the microbiota of healthy individuals. These MAGs were assigned to taxonomic clades that were also found in the microbiota of patients with Crohn's disease.

Domain	Acting on	Potential activities of interest
CBM4	AX	Xylanase
CBM35	AX	Xylanase
GH5 2	AX	Endo-b-1,4-xylanase
GH5 4	AX	Endo-b-1,4-xylanase
GH5 21	AX	Endo-β-1,4-xylanase
GH5 54	AX	Endo-b-1,4-xylanase
GH8	AX	Endo-β-1,4-xylanase
GH10	AX	Arabinoxylan-specific endo-β-1,4-xylanase
GH30 8	AX	Glucuronoarabinoxylan-specific endo-β-1,4-xylanase
GH120	AX	β-xylosidase
GH115	AX	Xylan α-1,2-glucuronidase
CE1	AX	Acetyl xylan esterase
CE2	AX	Acetyl xylan esterase
CE3	AX	Acetyl xylan esterase
CE4	AX	Acetyl xylan esterase
CE6	AX	Acetyl xylan esterase
CE7	AX	Acetyl xylan esterase
AA10	AX	Xylan oxidase
GH3	AX; pectin	Xylan 1,4-β-xylosidase; α-L-arabinofuranosidase
GH43 10	AX; pectin	Xylan 1,4-β-xylosidase; α-L-arabinofuranosidase
GH43 11	AX; pectin	Xylan 1,4-β-xylosidase; α-L-arabinofuranosidase
GH43 12	AX; pectin	Xylan 1,4-β-xylosidase; α-L-arabinofuranosidase
GH43 27	AX; pectin	Xylan 1,4-β-xylosidase; α-L-arabinofuranosidase
GH43 9	AX; pectin	α-L-arabinofuranosidase
GH43 16	AX; pectin	α-L-arabinofuranosidase
GH43 18	AX; pectin	α-L-arabinofuranosidase
GH43 19	AX; pectin	α-L-arabinofuranosidase
GH43 26	AX; pectin	α-L-arabinofuranosidase
GH43 33	AX; pectin	α-L-arabinofuranosidase
GH43 17	AX; pectin	α-L-arabinofuranosidase; endo-β-1,4-xylanase

GH43 34	AX; pectin	β-D-galactofuranosidase; α-L-arabinofuranosidase
GH98	AX; pectin	Endo-β-1,4-galactosidase; endo-β-1,4-xylanase
GH1	AX; pectin	β-galactosidase; β-xylosidase
GH2	AX; pectin	β-galactosidase; α-L-arabinofuranosidase; β-xylosidase
GH43 1	AX; pectin	β-galactosidase; α-L-arabinofuranosidase; β-xylosidase
CE12	AX; pectin	Acetyl xylan esterase; pectin acetylesterase
CBM51	Pectin	Galactosidase
GH42	Pectin	β-galactosidase
CBM32	Pectin	Polygalacturonase
GH28	Pectin	Exopolygalacturonase; rhamnogalacturonase
GH78	Pectin	α-L-rhamnosidase
GH105	Pectin	Unsaturated rhamnogalacturonyl hydrolase
CE8	Pectin	Pectin methylesterase
PL11 1	Pectin	Rhamnogalacturonan endo- / exolyase
PL26	Pectin	Rhamnogalacturonan exolyase
PL9 1	Pectin	Exopolygalacturonate lyase; rhamnogalacturonan endolyase

Table S5. Enzyme domains acting on arabinoxylan (AX), pectin and other carbohydrates (Other) an-notated in metagenome-assembled genomes (MAGs) recovered from the microbiota of patients with Crohn's disease. These enzyme domains were not found in MAGs recovered from metagenome samples from healthy individuals.

Domain	Acting on	Potential activities of interest
CBM54	AX	Xylanase
CBM42	AX; pectin	Arabinofuranosidase
PL4	Pectin	Rhamnogalacturonan endolyase
CBM20	Other	Starch-binding enzyme
CBM46	Other	Cellulase
CBM69	Other	Starch-binding enzyme
PL13	Other	Heparin lyase
PL15 2	Other	Heparin lyase
PL17 1	Other	Oligoalginate lyase

Table S6. Enzyme domains acting on arabinoxylan (AX), pectin and other carbohydrates (Other) annotated in metagenome-assembled genomes (MAGs) recovered from the microbiota of healthy individuals. These enzyme domains were not found in MAGs recovered from metagenome samples from patients with Crohn's disease.

Domain	Acting on	Potential activities of interest
GH11	AX	Endo-β-1,4-xylanase; exo-1,4-β-xylosidase
PL1 6	Pectin	Pectin lyase
PL9 2	Pectin	Pectin lyase
PL2 2	Pectin	Exo-polygalacturonate lyase
PL11 2	Pectin	Rhamnogalacturonan endo- / exolyase
PL4 4	Pectin	Rhamnogalacturonan endolyase
CBM21	Other	Starch-binding enzyme
CBM25	Other	Starch-binding enzyme
CBM63	Other	Cellulase
CBM68	Other	Pullulanase
GH13 33	Other	Trehalose synthase
GH23	Other	Lysozyme type G
GH48	Other	Cellobiohydrolase
GH5 48	Other	Chitosanase
GT87	Other	α-1,2-mannosyltransferase

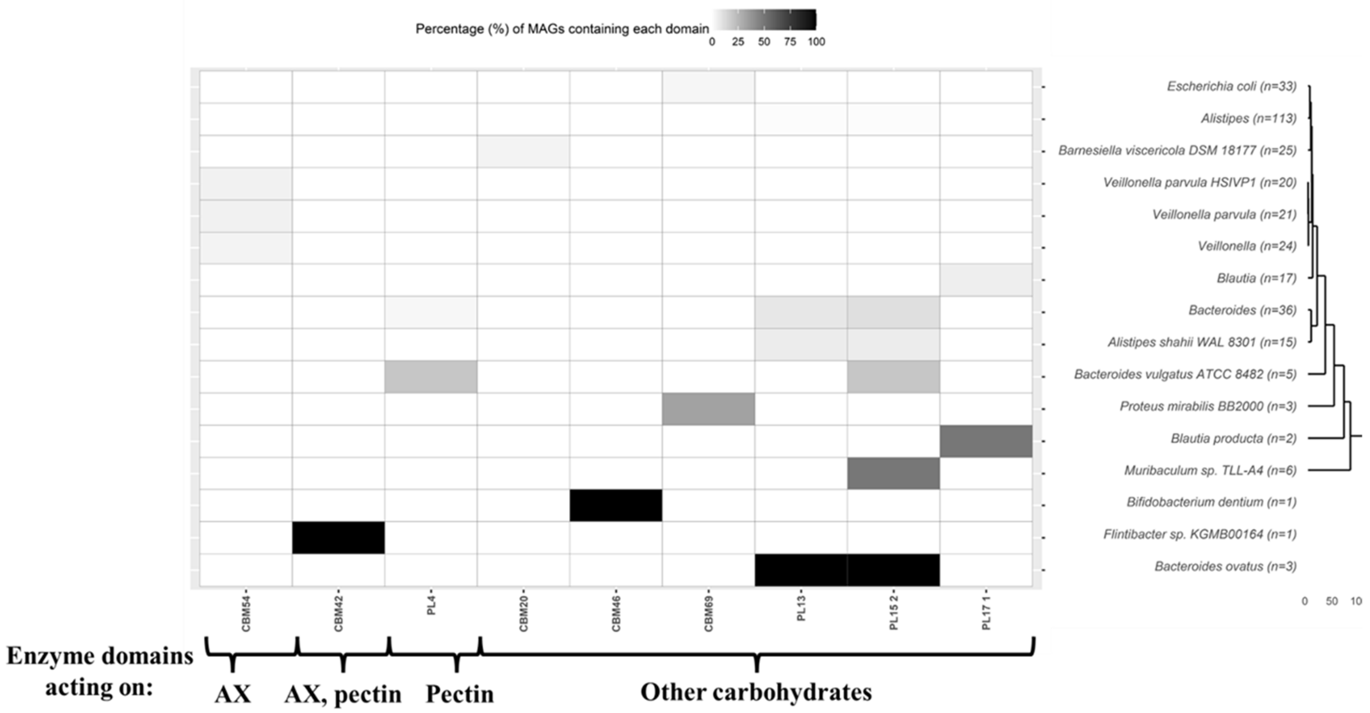

Figure S1. Heatmap showing the presence of different glycosidases (indicated as black cells) in metagenome-assembled genomes (MAGs) recovered only from the microbiota of patients with Crohn's disease (see Table S5). These enzyme domains were not found in MAGs recovered from metagenome samples from healthy individuals. The percentage (%) of MAGs containing each functional domain is shown. Specifically, glycosidases capable of degrading arabinoxylan (AX), pectin and other carbohydrates are illustrated. Glycosidase functional domains showing coverage values higher than 0.95 were annotated. Codes corresponding to the Carbohydrate-Active enZYmes Database (CAZy) family of each enzyme have been assigned.

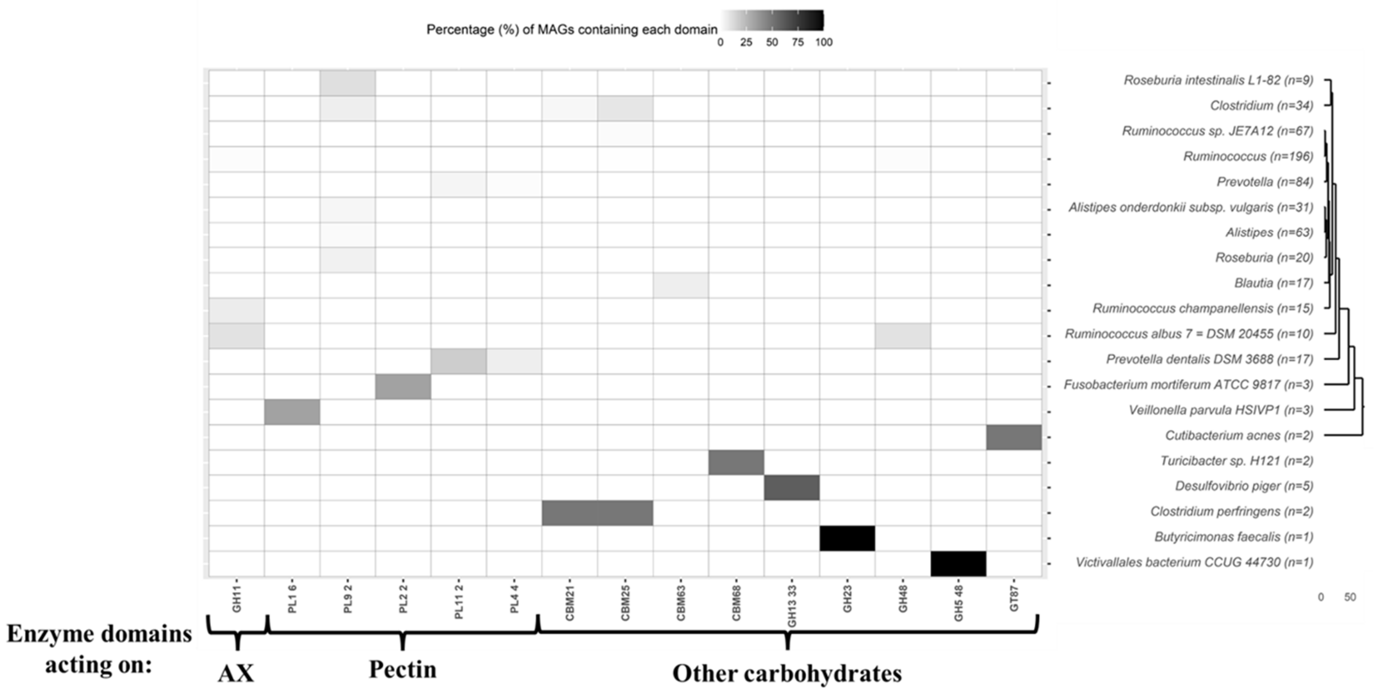

Figure S2. Heatmap showing the presence of different glycosidases (indicated as black cells) in metagenome-assembled genomes (MAGs) recovered only from the microbiota of healthy individuals (see Table S6). These enzyme domains were not found in MAGs recovered from metagenome samples from patients with Crohn's disease. The percentage (%) of MAGs containing each functional domain is shown. Specifically, glycosidases capable of degrading arabinoxylan (AX), pectin and other carbohydrates are illustrated. Glycosidase functional domains showing coverage values higher than 0.95 were annotated. Codes corresponding to the Carbohydrate-Active enZYmes Database (CAZy) family of each enzyme have been assigned.

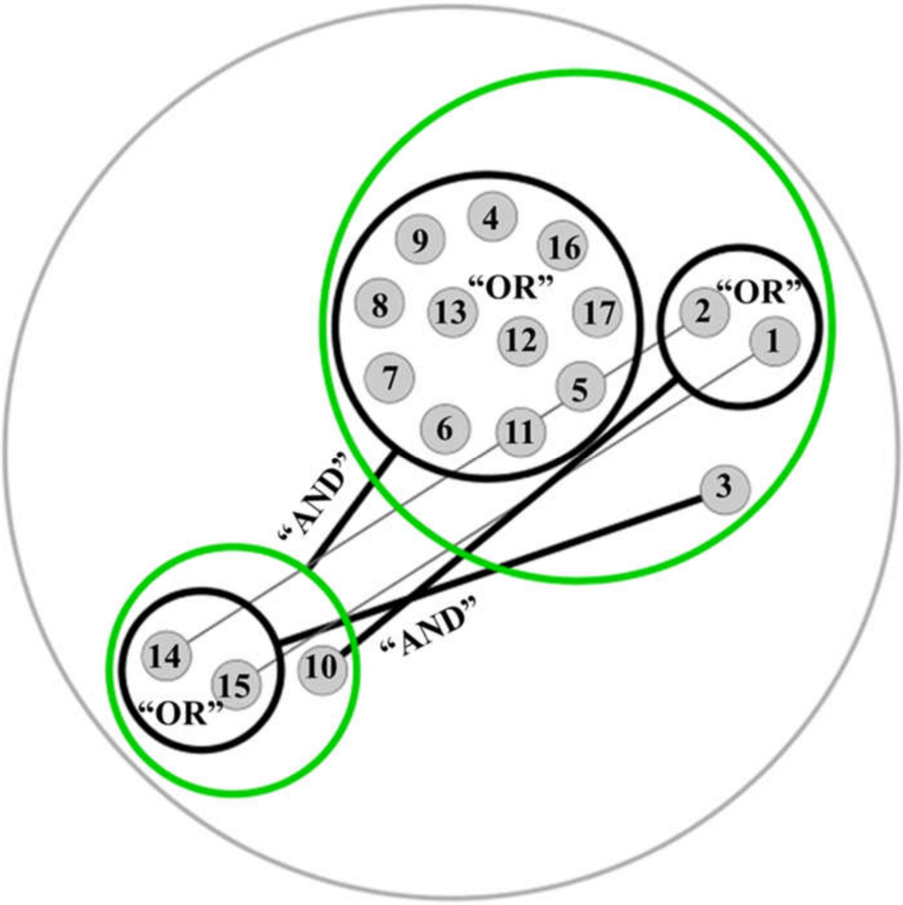

Figure S3. Metabolic network illustrating potential cross-feeding mechanisms between metagenome-assembled genomes (MAGs) recovered from the microbiota of healthy individuals in the presence of arabinoxylan. These MAGs involve beneficial arabinoxylan and pectin degraders (Akkermansia muciniphila, Barnesiella viscericola DSM 18177, Paraprevotella xylaniphila YIT 11841) that were also found in the microbiota of patients with Crohn's disease (see Figure 1). In addition, MAGs of other microorganisms that were not identified in the microbiota of patients with Crohn's disease and might be greatly affected by gut dysbiosis were also chosen. Network nodes (i. e. circles containing different microbial communities showing equivalent metabolic functions) are connected by black lines indicating synergistic relationships between communities and complementary metabolic functions. Metabolic functions of MAGs from different nodes are needed to achieve the maximum number of end-products from arabinoxylan as well as other colonic metabolites (this mutualistic relationship is indicated by the conjunction "AND"). MAGs inside the same node play the same role and could be replaced by other members from the same community (this similar role is indicated by the conjunction "OR"). 1: A. muciniphila, 2: B. viscericola DSM 18177, 3: P. xylaniphila YIT 11841, 4: unidentified Parabacteroides species, 5: unidentified Roseburia species, 6: Bifidobacterium adolescentis ATCC 15703, 7: Acidaminococcus fermentans DSM 20731, 8: Brachyspira murdochii DSM 12563, 9: Desulfovibrio piger, 10: Faecalitalea cylindroides T2-87, 11: Lactobacillus ruminis ATCC 27782, 12: Methanobrevibacter smithii ATCC 35061, 13: Streptococcus thermophilus, 14: Veillonella atypica, 15: Clostridioides difficile, 16: Lactobacillus mucosae LM1, 17: Veillonella parvula HSIVP1.
